# Huckleberry Finn’s Conscience: Reckoning with the Evasion

**DOI:** 10.1007/s10892-020-09341-3

**Published:** 2020-09-12

**Authors:** Steve Clarke

**Affiliations:** 1 School of Humanities and Social Sciences, Charles Sturt University, Bathurst, Australia; 2 Wellcome Centre for Ethics and Humanities & Uehiro Centre for Practical Ethics, Faculty of Philosophy, University of Oxford, Oxford, UK

**Keywords:** Huckleberry Finn, Nomy Arpaly, Jonathan Bennett, Conscious moral deliberation, the evasion, slavery

## Abstract

Huck Finn’s struggles with his conscience, as depicted in Mark Twain’s famous novel *The Adventures of Huckleberry Finn* (AHF) (1884), have been much discussed by philosophers; and various philosophical lessons have been extracted from Twain’s depiction of those struggles. Two of these philosophers stand out, in terms of influence: Jonathan Bennett and Nomy Arpaly. Here I argue that the lessons that Bennett and Arpaly draw are not supported by a careful reading of AHF. This becomes particularly apparent when we consider the final part of the book, commonly referred to, by literary scholars, as ‘the evasion’. During the evasion Huck behaves in ways that are extremely difficult to reconcile with the interpretations of AHF offered by Bennett and Arpaly. I extract a different philosophical lesson from AHF than either Bennett or Arpaly, which makes sense of the presence of the evasion in AHF. This lesson concerns the importance of conscious moral deliberation for moral guidance and for overcoming wrongful moral assumptions. I rely on an interpretation of AHF that is influential in literary scholarship. On it the evasion is understood as an allegory about US race relations during the 20-year period from the end of the US Civil War to the publication of AHF.

## Introduction

Huck Finn’s struggles with his conscience, as depicted in Mark Twain’s famous novel *The Adventures of Huckleberry Finn* (AHF) (1884), have been much discussed by philosophers; and various philosophical lessons have been extracted from Twain’s depiction of those struggles. Two of these philosophers stand out, in terms of influence: Jonathan Bennett (1974) and Nomy Arpaly (2002, 2003).^1^ Both focus, for the most part, on a sequence of events that takes place in Chapter 16. It involves Huck, a 13 or 14-year old white boy, who has been running away from his abusive, alcoholic father, and Jim, a black adult slave, who has been running away from his owner, Miss Watson. They have escaped from St. Petersburg, Missouri, the (fictional) town on the banks of the Mississippi river where both had been living; and they have been drifting down the Mississippi on a raft. When he thinks that they are nearing the city of Cairo, where the Ohio river joins the Mississippi, Jim becomes excited and declares that he will regard himself as free the moment they sight Cairo. As Cairo is in Illinois, where slavery was prohibited during the 1830s and 40s, the period in which AHF is set, Jim’s attitude is not unreasonable.^2^

Jim’s declaration provokes moral confusion in Huck, as he experiences the sensation of his conscience berating him for assisting Jim in running away from Miss Watson (1884: 97). Oblivious to the inner turmoil that he has unleashed in Huck, Jim announces plans to rescue his wife and two children from slavery. They are slaves on a farm near to Miss Watson’s home. Jim proposes to save enough money to buy first his wife and then his children out of slavery and, if their owner refuses to sell them, to get an ‘Ab’litionist’ to go and steal them and bring them to him (Twain 1884: 97–8). This announcement leads Huck to make a decision. According to him: “My conscience got to stirring me up hotter than ever, until at last I says to it, ‘Let up on me – it ain’t too late, yet – I’ll paddle ashore at the first light, and tell’” (Twain 1884: 98).

Huck takes a canoe, which he had found earlier, and starts to paddle ashore. However, he only travels 50 yards before he crosses paths with a skiff containing a pair of armed slave hunters who are searching for a group of runaway slaves. They identify Huck as coming from the raft and ask him if there is anyone else on it. Huck reports that there is one man on the raft. They then present Huck with the chance to act on his conscience by asking him if the man is black or white. If he tells them the man on the raft is black, then the slave hunters will go and capture Jim; and Huck’s conscience will be assuaged. Huck tries to do what he considers to be right, but:

… the words wouldn’t come. I tried, for a second or two, to brace up and out with it, but I warn’t man enough – hadn’t the spunk of a rabbit. I see I was weakening; so I just gave up trying, and up and says – ‘He’s white’. (Twain 1884: 99)

Huck returns to the raft:

… feeling bad and low, because I knowed very well I had done wrong, and I see it warn’t no use for me to try to learn to do right; a body that don’t get *started* right when he’s little, ain’t got no show – when the pinch comes there ain’t nothing to back him up and keep him to his work, and so he gets beat. (Twain 1884: 101)

After blaming his upbringing for what he perceives as his immoral behavior, Huck makes a disturbing decision which will have significant repercussions for both Huck and Jim. This is to forego the difficulty of consciously deliberating about the morality of his behavior in future:

Then I thought a minute and says to myself, hold on, – s’pose you’d a done right and give Jim up; would you felt better than what you do now? No, says I, I’d feel bad – I’d feel just the same way I do now. Well, then, says I, what’s the use you learning to do right, when it’s troublesome to do right and ain’t no trouble to do wrong, and the wages is just the same? I was stuck. I couldn’t answer that. So I reckoned I wouldn’t bother no more about it, but after this always do whichever come handiest at the time (Twain 1884: 101).^3^

Bennett portrays Huck’s behavior during this sequence of events as resulting from a contest between bad morality, expressed through his conscience, which has been shaped by the racist, slave-owning society he has grown up in, and his feelings of sympathy for Jim. Bennett declares sympathy the winner of the contest (1974: 126). He uses the term ‘sympathy’ very broadly, to refer to “every sort of fellow-feeling” (1974: 124). I will follow him in using the term this way. Bennett depicts Huck as someone whose sympathies run “wide and deep” (1974: 131). On this characterization, it is not particularly surprising that Huck’s sympathies would override his moral convictions and that he would find himself unable to turn Jim over to the slave hunters. The overall lesson Bennett (1974) extracts from AHF is that sympathy can and should play an important role in limiting the influence of bad moral presuppositions on behavior.^4^ While he does not argue that one should always defer to sympathy when it conflicts with moral presuppositions, Bennett does think it is important to place significant weight on one’s sympathies (1974: 133). He thinks this because he also thinks that we have the ability to exercise ‘ultimate control’ over the content of our moral principles, and according to him, “… one way such control can be exercised is by checking of one’s principles in the light of one’s sympathies” (1974: 132).

Arpaly considers the sequence of events of Chapter 16 in the context of Huck’s developing relationship with Jim, and she draws a different lesson than does Bennett. According to her, in their travels Huck “… constantly perceives data (never deliberated upon) that amount to the message that Jim is a person, just like him” (2003: 77). This message is flatly inconsistent with the racist presuppositions of the slave-owning society Huck has grown up in, which he endorses. Huck’s failure to hand Jim over to the slave hunters is explained by Arpaly as “… to a large extent the result of the fact that he has come to perceive Jim as a person, even if his conscious mind has not yet come to reflective awareness of this” (2002: 230). Huck is morally praiseworthy for helping Jim because he acts for reasons that are grounded in his recognition of Jim’s personhood, or so Arpaly argues (2002: 238). Arpaly acknowledges that Huck harbors unpraiseworthy racist attitudes. However, she declares Huck’s racism to be shallow, in contrast with the depth of his concern for Jim as a person (2002: 238).^5^

In my view, neither of the lessons that Bennett (1974) and Arpaly (2002, 2003) draw from AHF are supported by a careful reading of the book. This becomes apparent when we consider Chapters 33–43, the final part of AHF, which literary scholars often refer to as ‘the evasion’. In this part of the book Huck is joined by his friend Tom Sawyer and together they treat Jim in a way that is highly unsympathetic and far from praiseworthy. When we consider Huck’s behavior during the evasion it becomes apparent that his sympathies cannot be relied upon to limit the influence of his bad moral presuppositions on his behavior. Huck’s behavior during the evasion also throws into question whether he ever actually recognizes Jim’s personhood.

I’ll discuss the evasion in the next section of the paper. In Sect. 3 I’ll consider Huck’s sympathies in more detail, as well as discussing another problematic aspect of Bennett’s (1974) interpretation of AHF. In Sect. 4 I’ll turn my attention to Arpaly. I’ll argue that if we accept Arpaly’s account of what is involved in perceiving personhood, then, on the most plausible interpretation of AHF, Huck never perceives Jim as a person. In Sect. 5 I’ll consider whether or not Bennett’s and Arpaly’s misreading of AHF suggests anything more than an unfortunate choice of example. I’ll argue that in both cases it does. In Sect. 6 I’ll discuss an allegorical interpretation of the evasion, which is influential in literary scholarship. In Sect. 7 I’ll discuss a philosophical lesson that can be extracted from AHF, when we consider the book as a whole, which complements the allegorical interpretation of AHF. A brief conclusion follows Sect. 7. Throughout my discussion I’ll make the uncontroversial assumptions (also made by both Bennett and Arpaly) that racism and slavery are immoral. In addition, I will work with the not very controversial assumption that by the time Sam Clemens (Mark Twain is a *nom de plume*) completed AHF, approximately 20 years after the end of the US Civil War, he regarded both slavery and racism as immoral.^6^

## The Evasion

Between Chapters 19 and 31 Huck and Jim are joined in their travels by two conmen, the Duke and the King. The conmen eventually betray Jim, selling information about his whereabouts to slave hunters. Huck realizes what has happened and finds out that Jim is being held captive on a plantation owned by a local family, the Phelps, in advance of his owner being located (Twain 1884: 227–30). Huck then decides to try to free Jim, but not until he has gotten through another struggle with his conscience. This struggle involves him writing a note to Miss Watson, telling her where Jim is being held, and then tearing up the note. When Huck tears up the note he declares that he is willing to go to hell, if that is a price he has to pay to free Jim (Twain 1884: 230–2). The Phelps are related to Huck’s friend Tom Sawyer, who is on his way to visit them. Huck finds this out and intercepts Tom before his arrival at the Phelps’ plantation. Huck then asks Tom to help him free Jim. Huck does not actually expect that a well-brought up boy like Tom will participate in such a “dirty low-down business”, and is surprised, and somewhat appalled, when Tom readily agrees to help, reporting that Tom “… fell, considerable, in my estimation” (1884: 245).

Huck and Tom soon establish that Jim is locked inside a cabin on the Phelps’ plantation and that it would be very easy to free him (1884: 254). However, Tom wants them to drag out the freeing of Jim so that it follows something like the pattern of romantic novels about escaping prisoners—such as the novels of Walter Scott and Alexandre Dumas—that Tom is enamored with. Earlier on in the book, Huck had expressed disdain for Tom’s incessant make-believe game playing,^7^ and had resigned from ‘Tom Sawyer’s Gang’ (1884: 13), but this time he decides to go along with Tom’s plans. These include hiding a piece of a brass candlestick in Jim’s food, which when Jim bites into it, “… most mashed all his teeth out” (1884: 271), and filling Jim’s cabin with spiders, snakes and rats. Jim is repeatedly bitten by rats, deprived of sleep and generally traumatized (1884: 289). Tom also contemplates sawing one of Jim’s legs off, but eventually decides against it (1884: 260–1); and he declares his desire to keep ‘the evasion’ going for as long as 80 years (1884: 270–1).^8^

After 3 weeks of amusing himself at Jim’s expense, Tom starts to send out ‘nonnamous letters’ warning that an attempt to free Jim will soon take place (1884: 291–2). This provokes the formation of an armed posse of 15 local farmers. When they become aware of the existence of the posse Huck and Tom decide that they should try to free Jim immediately (1884: 294–6). They release Jim from the cabin and make their way to the raft. However, as they are escorting Jim to the raft Tom is shot in the calf. Jim is then recaptured, having waited for a doctor to arrive and tend to Tom’s wound. When he is recaptured some of the posse want to hang Jim, but they settle on giving him “a cuff or two, side the head, once in a while” (1884: 308). They also chain his hands and both legs—he had been unchained during the earlier period of imprisonment—and insist that he be fed nothing other than bread and water until his owner is located, or he is sold at auction. Previously, he had been fed a varied diet. After all of this, Tom reveals that he has known for some time that Jim had been set free in the will of his owner, Miss Watson, who died 2 months earlier (1884: 313).

Tom acts in an extremely callous manner towards Jim throughout the evasion. He allows Jim to remain imprisoned when he is legally free, and he colludes in the torture of Jim. It seems clear that Tom has hardly any sympathy for Jim, whom he regards as someone he is entitled to manipulate and abuse for his own entertainment. But what about Huck? Huck raises practical objections to some of Tom’s more elaborate plans, but he goes along with the mistreatment of Jim; and he displays no concern about the harms that he and Tom inflict on Jim. When Jim fails to notice the piece of brass candlestick hidden in his food and suffers serious damage to his teeth as a consequence of biting it, Huck expresses no sympathy for him whatsoever. Instead Huck expresses satisfaction, declaring that “there warn’t ever anything could a worked better” (1884: 271). When Huck finds out that Jim has been legally free for some time, and that Tom has been withholding this information, he does not condemn Tom for his utter disregard for Jim’s welfare. Huck’s only reaction is relief at having finally understood why someone with Tom’s upbringing would be willing to attempt to free a slave (1884: 314). Huck develops deep feelings of sympathy for Jim during their travels together. But very little of this sympathy is expressed during the evasion. Huck readily joins Tom in torturing Jim, and it fails to occur to Huck that their treatment of Jim is highly immoral.

## Bennett, Sympathy and Slavery

Bennett is right that sympathy plays an important role in shaping Huck’s behavior towards Jim and that during the sequence of events described in Chapter 16 it helps Huck to resist the demands of his conscience. But during this sequence of events limits to Huck’s propensity to feel appropriate sympathies are deftly revealed by Twain. When Jim explains that he intends to obtain the freedom of his wife and children, with the assistance of an abolitionist, if necessary, Huck’s reaction, which he never recants, is to condemn Jim. In Huck’s words: “I was sorry to hear Jim say that, it was such a lowering of him” (1884: 98). Often, when we feel sympathy for a person, we are also inclined to feel sympathy for those whom we know to be emotionally close to them. We understand that if one member of a family is suffering, other members of that family will suffer as a result, and our sympathy for the one member of a family is extended to other members. It should be obvious to Huck that Jim’s wife and children are suffering as a result of their separation from Jim and are deserving of some of the sympathy that he expresses for Jim. But Huck expresses no sympathy for them at all. If Jim and his wife and children were white then, in all likelihood, some of the sympathy that Huck expresses for Jim would be extended to his wife and children. But because Huck continues to think of Jim’s wife and children as property, and because his values are shaped by his endorsement of slavery, the sympathies that he would otherwise be disposed to feel for Jim’s wife and children are overwhelmed by his sense of disappointment that Jim is willing to violate the norms of their slave-owning society.

Not only does the sequence of events described in Chapter 16 reveal limits to Huck’s ability to feel appropriate sympathies, it also reveals a tendency he has to feel inappropriate sympathies. When Jim explains that he is willing to engage an abolitionist to steal his children out of slavery Huck expresses sympathy for their owner. To Huck, Jim’s children are “children that belonged to a man I didn’t even know; a man that hadn’t ever done me no harm” (1884: 98). Huck sympathizes with a white slave owner he doesn’t know who is in danger of being deprived of his human property. On this occasion, Huck’s sympathies do not protect him from the influence of bad morality—to adopt Bennett’s (1974) language. They reinforce bad morality.^9^

Because Huck continues to endorse the norms of a slave-owning society, and because he continues to think of Jim as a slave during the evasion, even when he is trying to free Jim, he fails to appreciate that hiding a piece of metal in Jim’s food, which causes significant damage to his teeth, and allowing Jim to be bitten by rats, is not a morally acceptable way to behave. Huck regards Jim sympathetically, but his sympathies do nothing to help Huck realize that he is colluding in the torture of a fellow human being. Contra Bennett, AHF is not a good illustration of sympathy operating as a check on moral principles. Often the sympathies that Huck expresses are shaped by his moral principles, and are unable, therefore, to serve as a reliable check on those principles.

Another problematic aspect of Bennett’s (1974) interpretation of AHF is his assumption that Huck is incapable of questioning the moral code of the slave-owning society that he has grown up in. According to Bennett (1974), “Huck clearly cannot conceive of having any morality except the one that he has learned—too late, he thinks—from his society” (1974: 131). Bennett also makes the following claim:

In his earliest years Huck wasn’t taught any principles, and the only ones he has encountered since then are those of rural Missouri, in which slave-owning is just one kind of ownership and is not subject to critical pressure. (1974: 125)

Even if we were to go along with Bennett (1974) and conclude that Huck is unable to conceive of a moral code in which slavery is considered wrong, we should not go along with him and conclude that Huck does not subject slave-owning to ‘critical pressure’. Moral disagreements concerning the proper treatment of slaves arose within the slave-owning society that Huck grew up in, and Huck takes sides on at least one and, quite likely two of these, thereby exposing the treatment of slaves by their owners to critical pressure. One such disagreement is about whether or not it is acceptable to separate families when selling slaves. Huck makes it clear that he strongly disapproves of sales of slaves that involve the separation of families (Twain 1884: 199). Another disagreement is about whether or not it is acceptable, all things being equal, to sell a slave if this involves imposing significantly harsher work conditions on that slave than they have hitherto experienced. Jim runs away from his owner, Miss Watson, because he overhears that she is about to sell him to a slave trader who is planning to take him “down to Orleans” and re-sell him (Twain 1884: 48). It was commonly accepted in Missouri at the time that the work conditions typically imposed on slaves down the river, where they often toiled on large plantations, were hasher than the conditions usually imposed on slaves in Missouri; and the harshness of these work conditions was often taken by white Missourans to be a *prima facie* reason to object to sales down the river (Jones 1992: 178; Dempsey 2003: 223).^10^

Huck’s belief about the *prima facie* wrongness of separating a family through the sale of a slave, as well as a belief that he is likely to have had, given that it was widespread in his community, about the *prima facie* wrongness of selling a Missouran slave to an owner who intends to put that slave to work on a plantation down the river, helps us to make sense of an interaction that takes place between Huck and Jim in Chapter 8. This is where Huck unexpectedly comes across Jim on Jackson’s island, in the middle of the Mississippi, not far downstream from St. Petersburg, and asks Jim what he is doing there. After Huck agrees not to tell anyone, Jim explains that he is running away from his owner (Twain 1884: 47–8). What is surprising is that Huck’s conscience lodges no objections to him sticking to this agreement until Chapter 16, even though, by the lights of the moral code that Huck accepts he is obliged to report Jim’s whereabouts to the relevant authorities at the first opportunity (1884: 47–8).^11^ Given that Huck persistently expresses the view that it is wrong to assist a slave who is running away from their owner, and given that he is well aware of the condemnation he is risking, from mainstream white Missouran society, by being seen as helping Jim to escape, it is very surprising that between Chapter 8 and Chapter 15 Huck appears to find it easy to stick to his agreement not to tell anyone that Jim has run away from his owner.^12^

When Jim first tells Huck that he is running away from Miss Watson he explains to Huck that he overheard that she was planning to sell him down the river (1884: 48). Huck is aware that Jim has a wife and children living near St Petersburg, so he is able to appreciate that this sale will involve separating Jim from his family, as well as, most likely, subjecting Jim to harsher work conditions. These considerations are salient to Huck at that point; and so, they act to silence his conscience—and this helps to explain why, in Chapter 8, he finds it easy to stick to his agreement not to tell anyone that Jim has run away from his owner. They are no longer salient to Huck in Chapter 16, when Jim raises the issue of his impending freedom, reveals his intention to try to free his enslaved wife and children, and re-unite with them, and thanks Huck for assisting him; and so, Jim’s declaration that he will soon regard himself as a free man provokes Huck’s conscience to voice its concerns.^13^

But we should not go along with Bennett and accept that Huck is unable to conceive of a morality in which slavery is considered wrong.^14^ To do so would be to demonstrate a failure to appreciate the context in which AHF is set. In the 1830s and 40s Missouri was the northernmost of the slave-owning states. The Mississippi river forms a natural border between Missouri and the free state of Illinois to the east. St. Petersburg, where Huck lives, is on the banks of the Mississippi. At the time abolitionists from Illinois and further east often crossed the river into Missouri to distribute pamphlets, and to assist slaves who wished to escape to one of the free states east of Missouri, or to Canada, via the ‘underground railway’. Meanwhile slave hunters patrolled the Mississippi and its banks (as we saw, Huck encounters one of these patrols), seeking to recapture escaped slaves, and vigilante mobs sought to disrupt the activities of abolitionists, sometimes lynching them.^15^ Even though Huck remains convinced, throughout AHF, that slavery is morally acceptable, it is hard to believe, given when and where he grew up, that he could be unaware that there is an alternative moral perspective, which has it that slavery is immoral.^16^ Like other Missourans of the time Huck would have been exposed to pro-slavery propaganda.^17^ Some of this emphasized the foolishness of abolitionists. Some of it alleged that many abolitionists had ulterior motives, seeking to encourage slaves to run away from their owners, so that they could be exploited by others.^18^ But even if Huck were to have formed the view that most abolitionists were frauds, he could not have failed to be aware that they promulgated an alternative moral view—abolitionism—which had it that slavery is immoral.

The text of AHF provides strong support for the conclusion that Huck understands, at least in rough outline, what abolitionism involves. When Jim first explains to Huck that he is escaping from his owner, Huck swears not to tell anyone, even though he is aware that he risks being labelled a “low down Ablitionist” and despised by other members of white Missouran society as a consequence (1884: 48). As we have already seen, in Chapter 16, Jim uses the term ‘Ab’litionist’ and expects that Huck will understand what he means. It might, perhaps be supposed that Huck doesn’t really understand what an abolitionist is, and just thinks that ‘abolitionist’ is a generic term of abuse. But this is unlikely. Huck appreciates that he is especially liable to be labelled an abolitionist if he fails to try to prevent Jim from running away from his owner; and Jim assumes that Huck understands that abolitionists are disposed to help slaves to escape from their owners.^19^

Does Huck really appreciate the future that sincere abolitionists have in mind for the slaves they assist? Huck is aware that he lives in a country divided between free states, in which slavery is illegal, and slave states and he is aware that abolitionists assist slaves to escape to free states. He uses the term ‘free state’ (Twain 1884: 89), and Jim uses the same term, presuming that Huck will understand what it means (Twain 1884: 94 and 97).There were free blacks living in Missouri at the time at which AHF is set, although they were far from equal to whites in the eyes of the law, and the freedoms they enjoyed were much more limited than those enjoyed by whites.^20^ So, it might perhaps be supposed that Huck believed that abolitionists only had in mind enabling blacks to move to places where they would enjoy the limited freedoms granted to free Missouran blacks at that time. But this is unlikely. Before he escapes from St. Petersburg, Huck is treated to a drunken rant by his father, ‘Pap’, after Pap encounters a black man from Ohio who, Pap discovers, is entitled to vote. Pap is outraged to find out that a black man is entitled to vote in his own country, and he vows never to vote again (Twain 1884: 30). After Pap’s rant Huck would appreciate (if he does not already) that at least some of the places that abolitionists help slaves to escape to are also places where blacks have rights that exceed the limited rights of free Missouran blacks.

## Arpaly, Personhood and Racism

Arpaly (2002, 2003) considers the phenomenon of inverse akrasia. When an agent performs an act that she should perform, despite her best judgment, she performs an inverse akratic act. Arpaly argues that some inverse akratic acts are morally praiseworthy, and she takes Huck’s failure to hand Jim over to the slave hunters in Chapter 16 of AHF, to be an example (2002: 227–8). Huck’s action is morally praiseworthy, according to Arpaly, because, despite his conscious racist beliefs, it is motivated by his unconscious recognition of Jim’s personhood. This is the reconstruction of Huck’s motives that Arpaly finds “most plausible” (2003: 76). According to her, the sustained interactions between Huck and Jim, during their travels, have led Huck to develop an unconscious perception of Jim’s personhood by Chapter 16 (2002: 229). Arpaly is confident that Huck has unconsciously recognized Jim’s personhood by Chapter 16, because she discerns evidence that Huck has already begun to unconsciously perceive Jim’s personhood in Chapter 15. This is where Huck finds himself unexpectedly apologizing to Jim, an action which Arpaly describes as “unthinkable in a society treating black men as subhuman” (2002: 229).

Arpaly’s standards for moral praiseworthiness are demanding. For an act to meet those standards it must be rightful and it must be performed for morally relevant reasons. She considers the case of Ron, a Jewish extremist who would like to kill Tamara, but refrains because Tamara is Jewish, and not because she is a person. Even though Ron acts rightfully he does not do so for morally relevant reasons. His reasons for acting are unconnected to the right-making features of his action, and so his action is not morally praiseworthy, according to Arpaly (2003: 74). Unlike Ron’s act, Huck’s act of refraining from handing Jim over to the slave hunters does meet Arpaly’s standards for moral praiseworthiness, she thinks, because it is performed “… because of its right-making features” (Arpaly 2002: 238).

Arpaly does not only refer to the ‘recognition of personhood’ to describe Huck’s developing unconscious attitude to Jim. She also writes that Huck “… begins to perceive Jim as a fellow human being” (2003: 77), and that “it is this awareness of Jim’s humanity that causes him to become emotionally incapable of turning Jim in” (2003: 10). Arpaly uses the language of personhood and of humanity interchangeably because she regards the recognition of personhood to involve the implicit recognition that being human makes one a person. The term ‘personhood’ is often used to indicate full moral status. A person is someone who has full moral status, whereas a non-person is someone or something that has either partial or no moral status. In white Missouran society in the 1830s and 40s white people were generally regarded as having full moral status and black slaves were generally regarded as having partial moral status. So, Arpaly is attributing a two-fold unconscious recognition to Huck in Chapter 16. Not only does he unconsciously recognize that his society is wrong about Jim lacking full moral status, Huck also unconsciously recognizes that his society is wrong about the basis for full moral status, which is humanity, rather than whiteness.

Huck’s behavior towards Jim during the evasion gives us reason to question Arpaly’s attribution to Huck of an earlier, unconscious recognition of Jim’s humanity. During the evasion Huck collaborates with Tom in torturing Jim and otherwise treating him inhumanely. It seems hard to believe that the good-natured Huck would participate in the torture of a person he regards as having full moral status, whom he had also befriended. So, his behavior suggests that he does not regard Jim as the moral equal of whites. Arpaly could perhaps respond to this line of criticism by conceding that Huck loses sight of Jim’s humanity during the evasion, but by insisting that nevertheless, Huck had recognized Jim’s humanity earlier on.

Another reason to question Arpaly’s attribution of a recognition of Jim’s humanity to Huck is Huck’s expressed attitudes towards slavery and slaves throughout AHF. At no point in AHF does it occur to Huck to doubt the moral acceptability of slavery. One might assume that someone who goes to considerable lengths to free a slave would express doubts about the moral acceptability of slavery, but Huck never does so.^21^ Not only does Huck never express doubts about the moral acceptability of slavery, he displays no particular concern for any of the dozen or so slaves, apart from Jim, whom he interacts with after Chapter 16.^22^ If Huck really were to have unconsciously recognized that humanity is an indicator of full moral status, rather than whiteness, by Chapter 16, then we should expect to see evidence of Huck’s improved treatment of black slaves other than Jim, after Chapter 16. However, the text of AHF does not supply such evidence. Arpaly might respond to these points by arguing that they show that Huck’s realization that humanity is a marker of full moral status is extremely slow to emerge.

I don’t wish to deny that Arpaly’s account of Huck’s moral development is possible to square with the text of AHF. I’ve been concerned to show that it rests on a strained interpretation of AHF, which does nothing to explain why Huck mistreats Jim during the evasion and why he remains committed to the rightness of slavery and unconcerned about the plight of any slaves other than Jim. I will now outline a rival account of Huck’s (truncated) moral development, which will explain these features of Huck’s attitudes and behavior. Because it can explain these features of Huck’s attitudes and behavior, and because Arpaly’s account cannot, it should be preferred to Arpaly’s account.

Throughout AHF Huck struggles to make sense of the inconsistencies he notices between the ways in which he has been brought up to expect that a black slave will behave and Jim’s actual behavior. For example, he is taught to believe that although blacks suffer somewhat when they are separated from family members, they do not suffer nearly as much as do white people. When he overhears Jim “moaning and mourning to himself” about being separated from his wife and children Huck makes the following observation: “… I do believe he cared just as much for his people as white folks does for their’n. It don’t seem natural, but I reckon it’s so” (1884: 169).

After Huck and Tom release Jim from the cabin on the Phelps’ plantation, and make their way to the raft, in Chapter 40, Huck observes another inconsistency between the behavior he has been taught to expect of a black slave and Jim’s actual behavior. Tom wants them to push off onto the river so they can continue their adventure, despite having a bullet lodged in his calf. But Jim refuses, insisting on waiting near Tom, while Huck goes to find a doctor, significantly reducing his chance to obtain lasting freedom thereby. This example of noble sacrifice for another is not what Huck has been brought up to expect of a black slave. All of a sudden it becomes clear to Huck that there is a compelling explanation for the discrepancies between the behavior he has been taught to expect of a black slave and Jim’s actual behavior. In Huck’s words: “I knowed he was white inside” (1884: 298).

Huck’s conclusion that Jim is ‘white inside’ seems to involve attributing a significantly higher level of moral status to Jim than was attributed, by white Missourans of the time, to ordinary blacks. But it does not involve a rejection of the presumption of black inferiority. Rather, it implies an affirmation of the inferiority of the vast majority of blacks who are not white inside. What Huck appears to be doing is revising the folk racial taxonomy that his society has furnished him with, to allow for an exception to the ordinary categorization of blacks. In order to explain to himself why a black person can behave in ways that are inconsistent with his society’s assumptions about blacks, he supposes that there is one, or perhaps a few anomalies to the taxonomy that underpins those assumptions. He reasons that these anomalies—the white inside—are deserving of superior moral status to ordinary blacks.

Allowing for exceptions is not uncommon in folk racial theorizing, which is unconstrained by the strict taxonomical norms of scientific theorizing. Apartheid South Africa recognized ‘honorary whites’ from various other countries, and of various races, and readily granted these honorary whites superior status to non-white South Africans. Also, Pino, a racist white character in Spike Lee’s 1989 film *Do the Right Thing*, is happy to reclassify several famous blacks, whom he admires, as ‘more than black’. In doing so he reconciles his admiration of them with his racist attitudes towards non-famous blacks.^23^

As Huck spends time travelling with Jim he finds himself experiencing increasing states of psychological discomfort. He feels impelled by the moral code that he has been brought up to accept to aid efforts to return Jim to his owner. He also feels impelled by his ever-deepening sympathies for Jim to refrain from assisting those efforts, and to help Jim to obtain his freedom instead. These days we might say, following Festinger (1957), that Huck experiences severe cognitive dissonance, as he is persistently driven to act in ways that are inconsistent with his deeply held beliefs. People who experience the sensation of cognitive dissonance are motivated to try to end that sensation, or at least to reduce its magnitude, either by modifying their behavior or by modifying their beliefs. For much of the time that Huck travels with Jim he finds himself unable to reduce cognitive dissonance. His two major crises of conscience, described in Chapter 16 and Chapter 31 of AHF, do not seem to help. When he enlists Tom’s aid, during the evasion, Huck experiences temporary respite from cognitive dissonance, as he is able to act as if he is freeing Jim, while actually collaborating with Tom to delay the freeing of Jim. The thought that Jim is white inside occurs to Huck at the end of the evasion and offers him more lasting respite. By modifying his beliefs about Jim and accepting the hypothesis that Jim is white inside, Huck can justify to himself his attempting to free Jim, while continuing to endorse his society’s view that blacks have a significantly lower moral status than whites and that, therefore, it is acceptable for whites to hold racist attitudes towards blacks, and to enslave blacks.

If the ‘white inside’ account of Huck’s moral development is accepted then we can explain why Huck goes to considerable lengths to free Jim, while remaining unconcerned about the plight of other black slaves and remaining committed to the rightfulness of slavery. We can also make sense of his inhumane treatment of Jim during the evasion. It does not occur to Huck that Jim is white inside until the end of the evasion. Arpaly could respond to me by denying the significance of Huck’s assertion that Jim is ‘white inside’, perhaps arguing that this is a confabulation Huck invents to explain his own behavior to himself, rather than an actual cause of that behavior. The problem with this line of response is that it would leave Arpaly without a good explanation of Huck’s treatment of Jim during the evasion and of Huck’s ongoing attitudes towards slavery and to slaves other than Jim.

Arpaly (2002, 2003) depicts Huck’s failure to hand Jim over to the slave hunters, in Chapter 16 of AHF, as a morally praiseworthy inverse acratic act. What are we to say about the moral praiseworthiness of this act on the white inside account of Huck’s moral development? To answer this question we need to try to reconstruct the unconscious reasoning that motivates the act. It may seem tempting to say that by Chapter 16 Huck has unconsciously concluded that Jim is white inside, and this is why he fails to hand Jim over to the slave hunters. But if we took this view we would find it difficult to explain Huck’s callous treatment of Jim during the evasion. What we should instead say, I think, is that by Chapter 16 Huck has unconsciously concluded that Jim is worthy of somewhat better treatment than the treatment typically given to blacks in his society; and so, he infers, Jim should not be handed over to the slave hunters. As Huck is aware, the slave hunters would probably turn Jim over to his new owner, and Jim would probably end up being subjected to harsh working conditions on a planation down the river, separated from his wife and children. By Chapter 16, Huck has determined that Jim does not deserve such poor treatment, but he has not concluded that Jim is worthy of significantly better treatment than the treatment typically given to blacks in his society. At that point Huck does not object to Jim remaining a slave. However, he thinks that if Jim is to remain enslaved, he ought to remain enslaved near St Petersburg, Missouri, where he can be together with his wife and children.^24^

If all that has unconsciously occurred to Huck by Chapter 16, is that Jim is worthy of somewhat better treatment than the treatment typically given to blacks in his society, then Huck’s act of refraining from handing Jim over to the slave hunters is not morally praiseworthy, on Arpaly’s (2002, 2003) account of moral praiseworthiness, because Huck’s reasons for action are unconnected to its right-making features. What would make it right for Huck to refrain from handing Jim over to the slave hunters, according to Arpaly, is for Huck to recognize that Jim is human, rather than subhuman. But, by Chapter 16, Huck has not unconsciously recognized that Jim is human rather than subhuman, to adopt Arpaly’s terminology. At that stage of AHF Huck still regards Jim as a subhuman, albeit one who is deserving of somewhat better treatment than most other subhumans. It may be, however, that I have underestimated the extent to which Huck has unconsciously recognized Jim’s ‘inner whiteness’, by Chapter 16. If so, then his action still does not meet Arpaly’s standards for moral praiseworthiness, because a recognition of inner whiteness is not connected to the right-making features of the act of refraining from handing Jim over to the slave hunters.

## Bennett and Arpaly Again

I have argued that once AHF is properly understood it becomes apparent it does not illustrate the points that either Bennett (1974) or Arpaly (2002, 2003) take it to illustrate. This is unfortunate for both authors, but it might be supposed that they could each respond to this state of affairs easily enough, by finding new examples to illustrate their respective points. Is there a problem for either or both authors that goes deeper than an injudicious choice of example? I believe that the ways in which AHF fails to illustrate their respective points reveals underlying conceptual difficulties for both authors. I’m not arguing that these are insurmountable, but they are significant and do need to be addressed. The difficulties revealed for Bennett (1974) are more significant than the ones revealed for Arpaly (2002, 2003). I’ll discuss the difficulties revealed for Bennett (1974) first.

Bennett (1974) argues that sympathy can serve as an important check on the influence of bad moral presuppositions on behavior (1974: 32). Having grown up in a slave-owning, racist society Huck is in the grip of bad morality and, as Bennett points out, Huck’s sympathy for Jim does help prevent him from acting in a way that would be wrongful—handing Jim over to slave hunters. However, sympathy does not prevent Huck from treating Jim cruelly during the evasion. And, as we have seen, a broader examination of Huck’s sympathies—towards the owner of Jim’s wife and children—and lack of sympathies—towards Jim’s wife and children—shows that these remain susceptible to the influence of bad morality. In illustrating the unreliability of sympathy as a form of protection against bad morality AHF makes trouble for Bennett (1974). He could probably find another literary example, to illustrate the power of sympathy, but this would not make AHF go away, and AHF paints a very compelling portrait of sympathy being influenced by bad morality. A different literary work, which depicted sympathy as exposed to the influence of bad morality, but unaffected by its influence, would be unlikely to feel as true-to-life as AHF.

It might be supposed that instead of appealing to literature to illustrate sympathy’s importance, as a form of protection against bad morality, Bennett could (and many would say should) appeal to findings in moral psychology instead. The problem with this suggestion, though, is that there is a significant strand of work in contemporary moral psychology, due to Bloom (2016), Prinz (2011) and others, that stresses the inability of our ordinary empathetic responses to promote consistent and appropriate moral behavior. These authors stress the extent to which our ordinary empathetic responses are shaped by prejudice and parochial ways of seeing the world. Twain’s careful depiction of Huck’s sympathies, as being shaped by the moral presuppositions of his unjust society, is consistent with the accounts of moral psychology favored by Bloom (2016) and Prinz (2011).^25^ Bennett could, of course, ignore these moral psychologists and just focus on the work of authors whose views are more congenial to him. But that would be to cherry pick the moral psychology literature.

Arpaly (2002, 2003) argues that Huck’s failure to hand Jim over to slave hunters in Chapter 16 of AHF is a morally praiseworthy inverse acratic act. I have disputed this conclusion, but I don’t wish to dispute that there can be morally praiseworthy inverse acratic acts. However, my reconstruction of the unconscious working of Huck’s mind, during the sequence of events in question raises an epistemic problem for Arpaly. I have argued that Huck’s moral development takes place more slowly than Arpaly supposes and follows a somewhat different trajectory to the one that she supposes it follows. Even if we just focus on the events described in AHF, up to and including Chapter 16, and set aside Huck’s behavior later in the book, there is no obvious reason to prefer Arpaly’s hypothesis that by Chapter 16 Huck has unconsciously recognized that Jim is a person, to my hypothesis that all that Huck has unconsciously recognized, by that stage of the book, is that Jim is worthy of somewhat better treatment than the treatment typically given to blacks in his society. Both hypotheses explain why Huck refrains from handing Jim over to the slave hunters in Chapter 16, equally well. This is a problematic situation for Arpaly. On her account, Huck is morally praiseworthy if he refrains from handing Jim over to the slave hunters because he is motivated by a recognition that Jim is a person, and he is not morally praiseworthy if he does so only because he is motivated by an unconscious recognition that Jim deserves somewhat better treatment than the treatment typically given to blacks in his society.

Without reliable information about the extent to which Huck’s unconscious perceptions of Jim have developed, by Chapter 16, and about the direction in which they are developing, we cannot establish whether or not Huck is morally praiseworthy for refraining from handing Jim over to the slave hunters. But such information would be hard to come by. The usual way in which we try to establish that particular unconscious mental states exist is by abductive inference. If postulated unconscious perceptions can be shown to be part of the best explanation of observed behavior then we can abductively infer to their existence. To abductively establish that particular unconscious perceptions exist we need to find grounds to rule out credible explanatory alternatives. Some of these alternatives will involve posits of similar unconscious perceptions which have developed to a lesser, or perhaps a greater extent, and which may be following somewhat similar, but nevertheless distinct developmental trajectories.

Because we don’t know all that much about how the unconscious mind functions, how it develops, and how it influences behavior, we will have difficulty finding good reasons to rule out posits of similar unconscious perceptions which have developed to a lesser, or perhaps a greater extent, and which follow somewhat similar but distinct developmental trajectories. So, we will have difficulty establishing which particular postulated unconscious perceptions figure in the best explanation of many given instances of behavior. We should be more cautious than Arpaly is, in attributing particular unconscious perceptions to people, and so more cautious than she is, in inferring that particular acts are morally praiseworthy inverse acratic acts.

## Evasion as Allegory

AHF is perhaps the most controversial book in American literature. Much of the debate about its literary merits centers on the issue of how to understand the relationship between the evasion and the rest of the book (Scott 2005: 187). Leo Marx is a noted critic of AHF. According to him,

… the ending of *Huckleberry Finn* makes so many readers uneasy because they rightly sense that it jeopardizes the significance of the entire novel. To take seriously what happens at the Phelps farm is to take lightly the entire downstream journey (Marx 1953: 425).

Huck’s finest moment occurs shortly before he arrives at the Phelps’ plantation, when he declares his willingness to risk going to hell in order to free Jim. From thereon Huck is a morally diminished character, turning a blind eye to the harms that he and Tom inflict on Jim.^26^ Because the evasion involves Huck effectively betraying Jim it makes for uncomfortable and, Marx (1953) thinks, unsatisfactory reading.^27^

AHF was first published in the mid-1880s, two decades after the US Civil war had ended and slavery had been abolished. One way of defending Twain’s inclusion of the evasion in AHF, which is highly influential in literary circles, is to interpret the evasion as an allegory about race relations in the US from the end of the US Civil War, up until the time of AHF’s initial publication (e.g. Budd 1962; Fishkin 2006a; Gollin and Gollin 1979; Holland 1982; Margolis 2001; Nilon 1992; Scott 2005).^28^ During this time period,^29^ many Southern state authorities had been doing what they could to ensure that blacks remained unequal to whites, and exploitable by them, first through the Black Codes of the late 1860s and then through ‘Jim Crow’ laws.^30^ Tom’s declared willingness to keep Jim imprisoned for as long as 80 years, even though he knows that Jim is legally free, fits very well with this interpretation.^31^

On the evasion-as-allegory reading of AHF, Twain can be understood as subtly parodying the unthinking attitudes of many well-meaning whites of the late 1860s, 1870s and early 1880s.^32^ It fails to occur to the well-meaning Huck, the narrator of AHF, that there might be anything morally problematic about slavery, even though he goes to great efforts to attempt to free a black slave, even though he is aware that there are US states in which slavery is illegal, and even though he is aware that there are people in his own country—abolitionists—who are actively opposed to slavery. Similarly, many otherwise well-meaning whites of the late 1860s, 1870s and early 1880s failed to think about the morality of the Black Codes and Jim Crow Laws, despite having gone through the trauma of the US Civil War and despite now accepting the wrongness of slavery (Gollin and Gollin 1979: 6). Twain can also be understood as warning his readers that they should not behave as Huck does during the evasion and acquiesce to the Tom Sawyers of the world, who are often to be found in positions of authority.^33^ They are not people who can be trusted to treat the former slaves fairly, despite what they may say.^34^

## The Importance of Conscious Moral Deliberation

If the evasion-as-allegory reading of AHF is correct, and Huck’s failure to deliberate about the morality of the slavery is analogous to the failure of many otherwise well-meaning whites of the late 1860s, 1870s and early 1880s to question the morality of the Black Codes and Jim Crow laws, then Huck is in a position to question the morality of slavery, but fails to do so. By showing us how easy it can be to fail to appreciate what the demands of morality are, when we fail to deliberate consciously about morality, AHF provides a striking illustration of a set of consequences that result from a failure to undertake conscious moral deliberation. Huck appears to come very close to questioning the morality of slavery. He realizes that it is wrong to allow a particular slave to remain enslaved, and he is motivated to go to great lengths to try to free that slave. Despite all this, he never pauses to reflect upon and deliberate about the morality of slavery itself.

It is worth considering a possible objection to my favored interpretation of AHF. This objection is that it could be argued that questioning the morality of slavery is never really a ‘live option’ for Huck, to use William James’ well-known terminology. Huck has been brought up in a society structured around institutionalized slavery and racism. Why think that he would be capable of questioning the moral appropriateness of institutions that went unquestioned by many members of his society?^35^ Huck’s easy assumption of black inferiority, which serves to underpin his commitment to slavery, is continually challenged by Jim’s behavior. By dint of his sustained association with Jim, Huck has been led to the realization that not every member of a race, whom his society regards as inferior and worthy of enslavement, is inferior and worthy of enslavement—hence his conclusion that Jim is white inside. This realization might well have sown enough doubt in Huck’s mind to motivate him to try to gather further evidence and testimony about the basis for his society’s assumption of black inferiority. He might have tried to see whether other blacks, apart from Jim, behaved in ways that were inconsistent with the model of black behavior endorsed by his society. He might also have tried to find out why abolitionists were convinced that slaves ought to be freed. It would not have been difficult for Huck to make contact with abolitionists. Abolitionists operated out of towns on the Illinois side of the Mississippi river, a short boat trip from Huck’s home town. Also, these abolitionists often visited Missouri, distributing pamphlets and attempting to help slaves escape from Missouri.^36^

There is no indication in AHF that it occurs to Huck to try to find out if other blacks, apart from Jim, behave in ways that are inconsistent with the model of black behavior endorsed by his society. Nor is there any indication that it occurs to him to try to find out more about the case for abolitionism. But it would not be difficult for Huck to do either of these things, and there is no reason to think that he would have any serious qualms about trying to do either. Huck is no coward and is capable of going against the norms of his society. He determines to try to set Jim free in Chapter 31, even though, like other members of his society he is convinced that attempting to free a slave is wrongful, and even though, he believes, that he will risk going to hell as a result of his actions.

It might perhaps be objected that I am expecting too much of a poorly educated teenage boy. But all that I am expecting is that he consider the possibility that his society’s endorsement of racism and slavery may be wrongful, when he is already aware that there are people who consider slavery to be wrongful—abolitionists—and when he is already aware that slavery is illegal in parts of his own country. Adolescents are sometimes stereotyped as selfish and incapable of moral reflection. However, the relevant evidence does not bear this stereotype out. Adolescents regularly make moral decisions and undertake moral reflection (Smetlana and Turiel 2006: 256). Also, adolescence is a time at which feelings of empathy and other pro-social feeling are increasingly associated with moral deliberation (Fabes et al. 1999: 11). Huck’s failure to deliberate about the morality of racism and slavery is unusual in an early adolescent who has seen, up close, the harms that racism and slavery have inflicted on a person with whom he empathizes. It would be unreasonable to condemn Huck for failing to realize that racism and slavery are immoral, but it is not unreasonable to fault him for failing to question the morality of racism and slavery.

Towards the end of the evasion, when the hypothesis that Jim is ‘white inside’ suddenly occurs to Huck and enables him to justify to himself his efforts to free Jim, while leaving the morality of the enslavement of the many blacks who are not ‘white inside’ unquestioned, Huck fails to reflect on that hypothesis and consider alternatives. This failure appears to have its origins in Huck’s decision, made in Chapter 16, to “bother no more about” conscious moral deliberation and to “always do whichever come handiest at the time” (Twain 1884: 101). A decision to continue to deliberate about morality would not have guaranteed that Huck would have realized that it was wrong of him and Tom to torture Jim, and nor would it have guaranteed that he would have realized that slavery and racism are wrong. However, it would have made it possible for Huck to experience these realizations.

The passage in Chapter 16, describing Huck’s first struggle with his conscience, is one of the most significant passages in AHF, but not for the reasons that either Bennett (1974) or Arpaly (2002, 2003) suppose. The decision Huck makes, at the end of the passage, which he sticks to, to give up on conscious moral deliberation, forecloses opportunities Huck may otherwise have taken to question the wrongfulness of some of the key assumptions guiding his behavior towards Jim, as well as towards other blacks. A philosophical lesson we can extract from AHF concerns the importance of conscious moral deliberation for moral guidance in general, and for overcoming wrongful moral assumptions in particular.^37^ It is not easy to realize that we have been making wrongful moral assumptions. We give ourselves almost no chance of realizing that we have been doing so if, like Huck, and the well-meaning whites of the late 1860s, 1870s and early 1880s, whom Twain appears to be parodying, we forego conscious moral deliberation.

## Conclusion

As we have seen, the philosophical lessons Bennett (1974) and Arpaly (2002, 2003) seek to extract from AHF are not supported by a careful reading of the book. This becomes particularly apparent when we consider the evasion: Chapters 33–43 of AHF. Contra Bennett (1974), the text of AHF does not provide support for the conclusion that we can rely on sympathy to limit the influence of wrongful moral assumptions on behavior; and contra Arpaly (2002, 2003), the text of AHF does not provide support for the conclusion that good unconscious perceptions can be relied on to countermand the influence of wrongful moral assumptions.

In order to try to extract their favored philosophical lessons from AHF Bennett (1974) and Arpaly (2002, 2003) both proceed by focusing on something that Huck doesn’t do. Huck conspicuously fails to hand Jim over to slave hunters, despite forming the intention to do so. I have also argued that there is a philosophical lesson that we can extract from AHF by focusing on something that Huck conspicuously doesn’t do. Despite being aware that he lives in a country in which many people consider slavery to be wrongful, and despite having seen up close the harms that racism and slavery have inflicted on a person with whom he empathizes–Jim–Huck fails even to consider the possibility that slavery and racism might be morally wrong. This failure has its origins in Huck’s earlier decision to forego moral deliberation. A general philosophical lesson that can be extracted from a careful reading of AHF concerns the importance of conscious moral deliberation for moral guidance. A specific philosophical lesson that can also be extracted concerns the importance of doing the hard work of consciously reflecting on and deliberating about our moral assumptions. If we fail to undertake this work we give ourselves almost no chance of identifying wrongful moral assumptions that we have been making, and then overcoming their influence on our behavior.

The specific and general philosophical lessons that can be extracted from AHF parallel specific and general political lessons that can also be extracted. The specific political lesson concerns the importance for whites in the US during the late 1860s, 1870s and early 1880s of consciously deliberating about the morality of the Black Codes and Jim Crow Laws. The general political lesson concerns the importance for majority groups of consciously deliberating about the morality of legal and institutional arrangements that impact on racial and other minority groups. This general political lesson is as important today as it was in Twain’s time.

## Footnotes

1 Other philosophers who have discussed AHF include Driver (2007), Fennell (2002), Gehrman (2018), Goldman (2010), Harris (1977), Kleist (2009), Lee (2009), Levy (2011), Markovits (2010), Montmarquet (2012), Rees (2006), Schinkel (2011), Taylor (2012), and Teichman (1975). Most philosophers who have discussed AHF regard Huck as morally admirable, all things considered. Dissenters include Schinkel, who stresses the extent to which deficiencies in Huck’s moral education limit his moral development (2011: 521–2), and Gehrman, who depicts Huck as ‘morally passive and thoroughly unreliable’ (2018: 126).

2 Slavery was prohibited in the Northwest Territory, part of which would become Illinois, by the Northwest Ordinance of 1787. Before the Dred Scott decision of the 1850s, American courts often took the view that slaves who set foot in free territory become permanently free (Bellamy 1973: 201).

3 Bennett interprets this passage as a declaration by Huck that he will “… give up morality altogether” (1974: 131). But the text does not support this interpretation. Huck resolves to do “whichever come handiest at the time”. If moral considerations happen to ‘come handiest’ then he will continue to act on these. What Huck resolves is to forego future attempts to reflect upon and deliberate about the moral considerations that occur to him. As AHF unfolds it becomes clear that many moral considerations occur to him, including a flurry of moral considerations that trigger a second major struggle with his conscience, in Chapter 31. The outcome of this struggle is that Huck defies his conscience and declares a willingness to go to Hell, if that is a price that he needs to pay, in order to free Jim from slavery (Twain 1884: 230–2). This is not a consequence that someone who had given up morality would willingly accept.

4 Because Bennett (1974) focusses on a sequence of events that take place in Chapter 16, it might be supposed that he is not actually concerned to interpret AHF accurately but is just selecting a passage from the book to make a point about the importance of sympathy. However, Bennett also discusses thoughts Huck has about conscience that appear in Chapter 33 (1974: 131). Furthermore, he makes broad claims about limits to Huck’s ability to conceive of morality (1974: 131), and about Huck’s upbringing (1974: 124). Clearly, Bennett takes his discussion of the sequence of events he focusses on to be informed by AHF as a whole.

5 Variants on the claim that Huck comes to perceive Jim as a person are made by Fennell (2002: 64), Taylor (2012: 589) and Crocker (2002: 64).

6 Clemens was openly racist as a young man and volunteered on the Confederate side in the US Civil War. He also expressed contempt for many abolitionists (Dempsey 2003: 231–8). However, he married into a prominent abolitionist family in 1870, and sought to make amends for his earlier behavior by anonymously paying for the education of a black student at Yale Law School. Clemens described this action as “part of the reparation due from every white to every black man” (Gollin and Gollin 1979: 6). He also donated money to Lincoln University to provide scholarships for black students, and supported the training of a black artist, paying for his studies in Europe (Pettit 1974: 125–6). For discussion of the evolution of Clemens’ thinking about race, in relation to the development of AHF, see Fishkin (2002, 2006a).

7 Chapter 3 concludes with Huck informing us that: “… I judged that all that stuff was only just one of Tom Sawyer’s lies. I reckoned he believed in the A-rabs and the elephants, but as for me I think different. It had all the marks of a Sunday school” (Twain 1884: 16).

8 The evasion is referred to by literature scholars as ‘the evasion’ because of the way Tom Sawyer describes what he is planning. In Tom’s words: “When a prisoner of style escapes, it’s called an evasion” (Twain 1884: 291).

9 Schinkel (2011) also argues, contra Bennett (1974) that Huck experiences misplaced sympathies. He points out that Huck expresses misplaced sympathy for Miss Watson, when his conscience berates him for failing to speak up about the fact that her slave, Jim, has run away. See Schinkel (2011: 512).

10 Jim is eventually freed because his elderly owner, Miss Watson, becomes ashamed of having attempted to sell him down the river and frees him in her will (Twain 1884: 313).

11 According to Twain: “… In those old slave-holding days the whole community was agreed as to one thing – the awful sacredness of slave property. To help steal a horse or a cow was a low crime, but to help a hunted slave … or hesitate to promptly betray him to the slave catcher, when opportunity offered was a much baser crime, & carried with it a stain, a moral smirch, which nothing could wipe away.” Cited in Beaver (1987: 4).

12 Perhaps it is not as easy for Huck to stick to his agreement during this period as appearances suggest, however. According to Schmidt, Huck undergoes a crisis of racial identity between Chapters 8 and 16, which can be discerned by carefully examining details of Huck’s interactions with Jim in this part of AHF (2003: 38–40).

13 It may seem odd that Huck does not question Jim about his future plans regarding his wife and children, in Chapter 8, when Jim first reveals to Huck that he has run away from his owner. Huck probably assumes, in Chapter 8, that Jim no longer has the option of being reconciled with his family who are enslaved in Missouri. Jim’s only options are to be sold down the river to a Southern slave state or to escape to a free state. Huck is caught off guard by Jim’s announcement of a plan to either buy or steal his family out of slavery in Chapter 16, but Huck does not cease hoping, for Jim’s sake, that Jim and his family may be reunited legally. Indeed, in the lead up to his second major struggle with his conscience, in Chapter 31, Huck contemplates a half-thought-through plan to have Jim re-enslaved in St Petersburg, Missouri so that Jim can be reunited with his family (1884: 230).

14 Unfortunately, other authors have gone along with Bennett (1974). These include Goldman (2010) and Rees (2006).

15 For discussion of the pre-Civil War contest, in Missouri, between abolitionists and proponents of slavery, see Dempsey (2003, Chapters 6 to 8).

16 Almost all abolitionists thought that slavery was morally wrong. However, not all thought that this was primarily because of injustices towards and harms committed against slaves. Some abolitionists were primarily concerned about the effects that slavery had on free, poor whites. Competition from slave labor drove down the wages of poor, unskilled whites. Others were primarily concerned with the effects that owning slaves had on the work ethic of slave owners. For further discussion, see Ferrell (2006).

17 For discussion, see Dempsey (2003, Chapters 16 and 17).

18 For illustrations, see Dempsey (2003: 198 and 213).

19 It could perhaps be argued that even though he is aware of an alternative moral viewpoint from which slavery is considered immoral, Huck is unable to conceive of this alternative as a ‘live option’ for him—to use William James’ well-known terminology. I’ll discuss this line of argument in Sect. 7.

20 Approximately 2.7% of blacks living in Missouri in 1840 were free (Bellamy 1973: 200). For discussion of the (very limited) rights enjoyed by free blacks in Antebellum Missouri, see Bellamy (1973).

21 As we have seen, Huck does express his feelings about the wrongness of separating families through the sale of a slave (Twain 1884: 199). But this sentiment was widespread throughout white Missouran society of his time and is entirely compatible with holding that slavery is morally acceptable.

22 Goldman also notes the inconsistency between Huck’s sympathy for Jim and his lack of sympathy for other black slaves (2010: 12–14).

23 See http://www.imdb.com/title/tt0097216/trivia?tab=qt&ref_=tt_trv_qu (accessed 25 February 2020).

24 This appears to remain Huck’s view even during his second crisis of conscience, in Chapter 31. During that crisis he reasons to himself that “it would be a thousand times better for Jim to be a slave at home where his family was, as long as he’d *got* to be a slave” (Twain 1884: 230). See also footnote 13.

25 Is Huck’s emotional reaction to Jim empathetic as well as sympathetic? The distinction between sympathy and empathy is often drawn as follows: When someone sympathizes with another they feel for them. When they empathize with them, they imagine themselves “in the other person’s shoes” (Prinz 2011: 212). Most philosophers interpret Huck as merely sympathetic towards Jim. However, Kleist interprets Huck as empathizing with Jim (2009: 261).

26 As Marx notes, Jim is similarly diminished, as he meekly submits to Tom and Huck’s game playing (1953: 429–30). Black literary critics have often condemned Twain’s depiction of Jim’s ready acquiescence to Tom and Huck’s game-playing during the Evasion. Henry describes it as “sinister and malicious” (1992: 37). Jones is also highly critical (1992: 186). For a defense of Twain’s characterization of Jim, see Fishkin (2002, 2006b).

27 Ernest Hemingway famously advised readers of AHF to stop at the point where Jim is sold out by the Duke and the King (Hemingway 1935).

28 For a survey of various different literary interpretations of the evasion, and of its relationship to the main body of AHF, see Henrickson (1993).

29 The period immediately after the Civil War is often referred to in the US the ‘Reconstruction Era’. I have not used this term because the Reconstruction Era is usually thought of as ending in 1877, 7 years before the publication of AHF.

30 The Black codes were racially discriminatory laws enacted in Southern States after the US Civil War. They were overridden by the Fourteenth and Fifteenth amendments to the US Constitution, in 1868 and 1870 respectively. The Black Codes were replaced in those same states with muted variants, which are often referred to as Jim Crow laws. See Leonard, Tenney and Davis (eds.) (1992: 58–9).

31 Tom’s declared willingness to continue the imprisonment of Jim for as long as 80 years is prescient. It would not be until 80 years after the publication of AHF that the US Federal Government overrode the Jim Crow laws, through the Civil Rights Act of 1964 and the Voting Rights Act of 1965.

32 Fishkin suggests that even the ludicrousness of Tom’s treatment of Jim during the evasion can be understood as Twain’s parodying of the sheer insanity of some aspects the treatment of blacks in the late 1860s, 1870s and early 1880s, such as granting previously unenfranchised blacks the right to vote but then ensuring that most are unable to exercise that right (2006a: 20).

33 It has been suggested to me that, given their stark differences in social standing, Huck has no choice but to acquiesce to Tom’s authority during the evasion. However, Huck had already rejected Tom’s authority early on in AHF, resigning from ‘Tom Sawyer’s Gang’ (Twain 1884: 13). When Huck defers to Tom during the evasion it is because doing so suits his purposes; and not because he is incapable of acting otherwise.

34 Johnston argues that Twain’s description of Jim’s acquiescence to Tom, during the evasion, is intended as a message for black readers, cautioning them against deferring to white Southern authorities (2013: 68).

35 One Missouran who questioned the morality of slavery was Sam Clemens’ older brother Orion, who became convinced that slavery was immoral before the start of the US Civil War (Budd 1962: 6).

36 See Dempsey (2003, Chapter 4), for examples of their activities.

37 The importance of conscious moral deliberation is often stressed by rationalist philosophers. For a recent defense of rationalism about morality, see May (2018).
